# Effects of Corn Straw Returning Patterns on Soil Bacterial Community Structure in Soybean Under a Corn-Soybean Rotation System

**DOI:** 10.3390/plants15070990

**Published:** 2026-03-24

**Authors:** Xiaohui Wang, Demin Rao, Debin Yu, Tong Cheng, Jing Zhao, Minghao Zhang, Fangang Meng, Wei Zhang

**Affiliations:** Jilin Academy of Agricultural Sciences (Northeast Agricultural Research Center of China), Changchun 130033, China

**Keywords:** corn straw returning, bacterial community structure, corn–soybean rotation system

## Abstract

Straw returning is an effective means of improving soil structure and increasing soil organic matter content. However, few studies have been conducted on the effects of corn straw returning on the soil microorganism community in soybean crops. In this paper, taking conventional combined tillage (CT) as a control, the effects of no-tillage with straw mulching (NTS), no-tillage with stubble retention (NT), and deep plowing with straw incorporation (DT) on soil bacterial community under a corn–soybean rotation system were studied. The results showed that the contents of soil total nitrogen, total phosphorus, available phosphorus, the activities of soil urease and acid phosphatase, and soil bacterial richness and diversity in the NTS treatment were significantly higher than those in other treatments. Moreover, the NTS treatment increased the abundance of *Acidobacteriota* and MND1 (unclassified bacterial genus) in the soil. The number of unique OTUs in the NTS treatment was the greatest (26.67%), with that of the CT treatment being the smallest (7.22%). Redundancy analysis (RDA) revealed that soil total nitrogen, total phosphorus, and available phosphorus are the key driving changes in bacterial community. Consequently, NTS treatment was the optimal approach for both soil fertility improvement and bacterial community optimization. This approach combines straw mulching and no-tillage, which not only exerts the nutrient supply effect of straw but also reduces the impact of soil disturbance on microbial habitats.

## 1. Introduction

With the continuous increase in corn yields in Northeast China, the amount of corn straw has also gradually risen, making the return of straw to the field an important method for its utilization [1,2]. Straw returning can improve soil structure, increase soil organic matter content, enhance soil fertility, reduce chemical fertilizer application, decrease environmental pollution, and maintain crop yield [3,4]. At present, the main straw returning approaches in production include straw mulching and straw deep plowing. Different straw returning methods exert significantly different effects on soil physical and chemical properties and soil microorganisms [5,6]. Researchers have found that straw incorporation through plowing promotes the accumulation of total soil organic carbon, whereas straw mulching favors the accumulation of active organic carbon [7,8]. However, straw incorporation through plowing can destroy the original soil structure, impact the formation and stability of soil macroaggregates, and accelerate the mineralization of soil organic matter. No-tillage, reduced tillage, and other conservation tillage methods cause less disturbance to the soil and reduce damage to soil aggregates; however, they may lead to light shading and reduced soil temperature, which are unfavorable in low-temperature spring years [9,10].

As the most active component in agroecosystems, soil microorganisms play a crucial role in straw degradation and resource utilization, serving as sensitive bioactivity indicators reflecting the effects of straw returning on soil. It has been reported that numerous microorganisms, such as bacteria, fungi, and actinomycetes, are involved in straw degradation. Among them, bacteria account for 70–90% of total soil microorganisms and play an important role in the decomposition of straw cellulose [11,12]. Utilizing microorganisms in straw provides optimal environmental conditions that promote straw degradation. Similarly, straw returning can supply sufficient carbon and nitrogen sources and energy for the growth and reproduction of microorganisms, thereby improving their diversity, abundance, and activity in the soil. Research has shown that straw returning not only increases the number of soil microorganisms in soil but also significantly affects soil microbial community structure, thus effectively promoting straw decomposition and transformation [13,14,15].

Soybean is an important dual-purpose crop, providing both grain and oil, and is valued for its protein and oil content. It not only serves as an important source of high-quality plant protein but also as a major raw material for edible oil production. Soybean oil accounts for 20% of global total edible oil output, and soybean also provides roughly two-thirds of the concentrated protein used for global livestock and poultry breeding. As the world’s fourth-largest soybean producer, China imported 105 million tons of soybeans in 2024, with domestic soybean output standing at a mere 20.65 million tons. At present, China has become one of the world’s largest soybean importers, with Brazil and the United States as the main import sources [16]. China’s low soybean self-sufficiency capacity is attributed to two main factors: first, the large planting area of corn results in limited land for soybean cultivation; second, as the main soybean-producing area in China, the black soil region in Northeast China suffers from severe black soil degradation, leading to a reduction in soil organic matter, a decrease in water retention capacity, and the weakening of nutrient regulation capacity, thus restricting the increase in soybean yield.

Straw returning is an effective method to resolve soil compaction, fertility decline, and soil degradation and to improve soybean yield per unit area. In one study, long-term corn straw returning significantly increased soil total phosphorus content, reduced the percentage of stable soil phosphorus, improved soil phosphorus availability and phosphorus supply capacity, and enhanced the storage of soil active carbon and nitrogen pools compared with straw removal [17]. In another study, researchers found that straw returning treatments significantly increased the average contents of soil organic carbon and total nitrogen by 16.78% and 16.23%, respectively, and the contents of microbial biomass carbon, ammonium nitrogen, and microbial biomass nitrogen by 0.24%, 7.23%, and 8.94%, respectively [18]. Straw returning has shown positive effects on soybean agronomic traits and the absorption of nitrogen, phosphorus, and potassium nutrients, increasing soybean yield, with this yield-increasing effect remaining stable during the two-year experimental period [4]. Straw returning has a significant promoting effect on the activities of soil urease, invertase, and catalase [19]. It can increase the relative abundances of cellulose-degrading fungi, saprophytes, and nitrogen cycle bacteria in the soil of soybean and reduce the relative abundance of pathogenic fungi [13].

In a large number of studies on straw returning approaches in China, researchers have focused on the impacts on the growth of corn and wheat, with few researchers investigating the changes in the soil microbial community of subsequent soybean crops under different corn straw returning approaches. To address the issue of severe soil degradation caused by long-term continuous corn cropping and resource waste due to excessive straw production in Northeast China, we based our study on a long-term fixed-position experiment of different straw returning approaches under a corn–soybean rotation system. We explored the effects of no-tillage with straw mulching, no-tillage with stubble retention, and deep plowing with straw incorporation on the bacterial diversity in the bulk soil of subsequent soybean crops, aiming to clarify the relationships among straw returning, soybean soil nutrients, soil enzymes, and bulk soil bacteria. We intended to screen the optimal straw returning approach suitable for long-term application in Northeast China to provide a reference for soybean yield increase and black soil protection.

## 2. Results

### 2.1. Analysis of Soil Nutrients and pH

The contents of soil total phosphorus and available phosphorus in the NTS treatment were significantly higher than those in the other three treatments (*p* < 0.05). Compared with NT, CT, and DT, the soil available phosphorus content in the NTS treatment was 80.89%, 108.15%, and 83.88% higher, respectively, and the soil total phosphorus content was 34.15%, 30.95%, and 30.95% higher, respectively. No significant difference was detected in soil available phosphorus content among the NT, CT, and DT treatments (*p* > 0.05). The soil total phosphorus content in the CT treatment was significantly lower than that in the NT and DT treatments, with values 13.06% and 11.67% lower, respectively. The soil total nitrogen content in the NTS treatment was significantly higher than that in the other three treatments (*p* < 0.05), with values 20.76%, 17.80%, and 19.50% higher than that in the NT, CT, and DT treatments, respectively; no significant difference was found in soil total nitrogen content among the NT, CT, and DT treatments (*p* > 0.05). The soil pH in the CT treatment was significantly lower than that in the DT treatment (*p* < 0.05), and no significant difference was observed between the CT, NTS, and NT treatments. In addition, no significant differences were observed in soil total potassium content, available potassium content, alkali-hydrolyzable nitrogen content, and organic matter content among all treatments (*p* > 0.05) (Table 1).

### 2.2. Analysis of Soil Enzyme Activities

The soil enzyme activities in all straw returning treatments were higher than those in the CT treatment; however, the effects of different straw returning approaches on soil enzyme activities differed significantly. The soil urease and acid phosphatase activities in the NTS treatment were significantly higher than those in the other three treatments. In the NTS treatment, urease activity was 8.67%, 18.48%, and 7.27% higher than that in the NT, CT, and DT treatments, respectively, and acid phosphatase activity was 18.28%, 36.58%, and 19.42% higher than that in the NT, CT, and DT treatments, respectively. No significant differences were observed in soil urease and acid phosphatase activities between the NT and DT treatments, with the activities of both being significantly higher than those in the CT treatment. Compared to the CT treatment, the urease activity in the NT and DT treatments was 9.03% and 10.45% higher and acid phosphatase activity was 15.48% and 14.37% higher, respectively.

**Figure 1:**
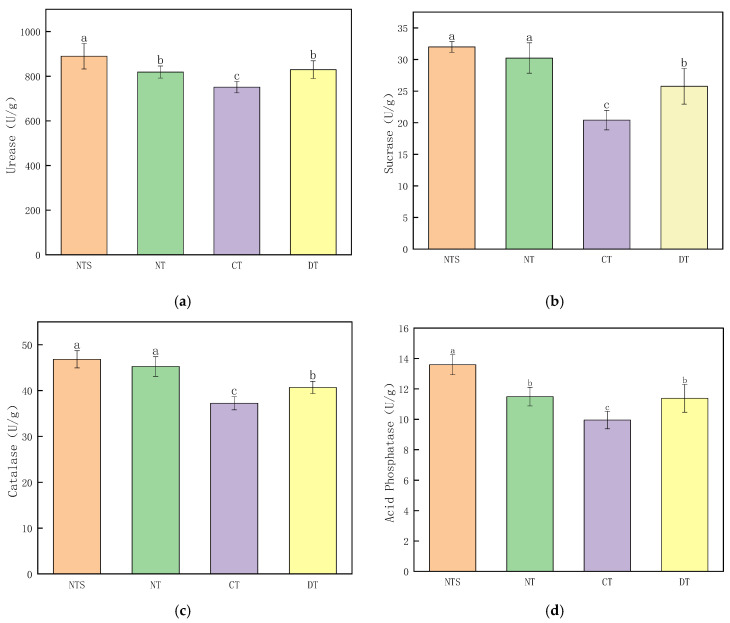
Soil urease activity (**a**), sucrase activity (**b**), catalase activity (**c**), and acid phosphatase activity (**d**) in soybean fields under different straw returning patterns. Different lowercase letters represent signiﬁcant differences among all treatments based on Duncan’s test (*p* < 0.05).

No significant difference was observed in soil sucrase and catalase activities between NTS and NT treatments; however, both were significantly higher than those in the DT and CT treatments. The sucrase activity in the NTS treatment was 56.69% and 24.15% higher than that in the CT and DT treatments, respectively, and the catalase activity was 25.79% and 15.09% higher than that in the CT and DT treatments, respectively. The sucrase activity in the NT treatment was 48.11% and 17.35% higher than that in the CT and DT treatments, respectively, and the catalase activity was 21.60% and 11.26% higher than that in the CT and DT treatments, respectively. Soil sucrase and catalase activities in the DT treatment were significantly higher than those in the CT treatment, with increases of 26.21% and 9.29%, respectively (Figure 1).

### 2.3. Differences in Soil Bacterial Alpha Diversity Analysis

**Figure 2:**
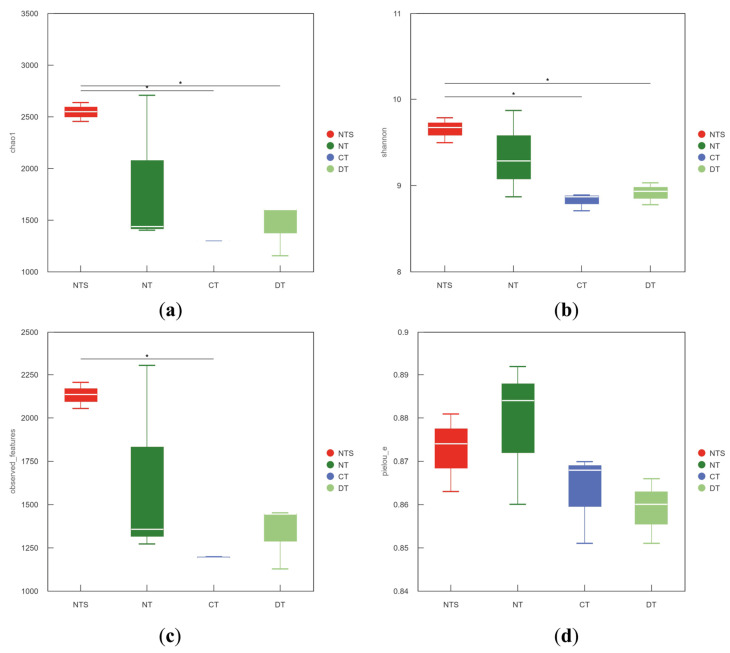
Soil bacterial Chao 1 index (**a**), Shannon index (**b**), observed features index (**c**), and Pielou_e index (**d**) in soybean fields under different straw returning patterns. * indicates a signiffcant level at *p* < 0.05.

Alpha diversity refers to the diversity within a specific environment or ecosystem, primarily reflecting the richness and evenness of species. In this study, the Chao1 and Observed features indices during Alpha diversity analysis were used to characterize species richness, the Shannon index for species diversity, and the Pielou index for species evenness. Significant differences were observed in the Chao1 and Shannon indices among all treatments at the filling stage. Specifically, the Chao1 and Shannon indices in the NTS treatment were significantly higher than those in the CT and DT treatments, and the Observed features index in the NTS treatment was significantly higher than that in the CT treatment. The Chao1 index in the NTS treatment was 2.09 and 1.86 times that in the CT and DT treatments, respectively; the Shannon index was 1.10 and 1.08 times that in the CT and DT treatments, respectively; and the Observed features index was 1.16 times that in the CT treatment. These results indicated that the NTS treatment possessed the highest soil bacterial richness and diversity. The Pielou index in the NTS and NT treatments was higher than that in the CT and DT treatments, with no significant differences among all treatments (Figure 2).

### 2.4. Analysis of Soil Bacterial Gene Abundance and Community Composition

**Figure 3:**
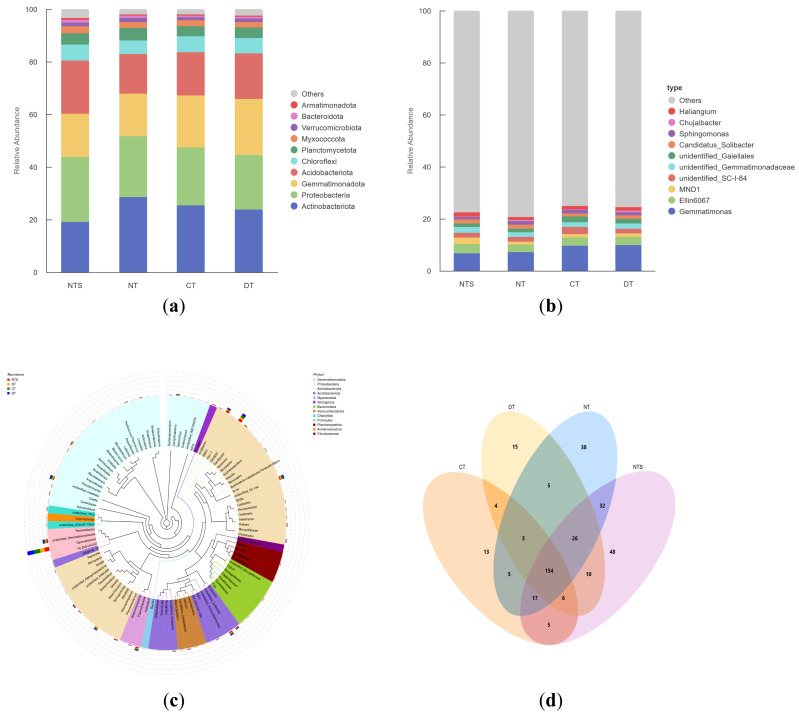
Bacterial Gene Abundance at the phylum (**a**) and genus (**b**) levels, phylogenetic tree (**c**), and Venn diagram (**d**) in soybean fields under different straw returning patterns. Numbers in overlapping regions represent the number of shared ASVs among different treatments, while numbers in non-overlapping regions represent the number of unique ASVs for each treatment.

At the phylum level, soil bacteria under different straw returning methods were dominated by *Actinobacteriota*, *Proteobacteria*, *Gemmatimonadetes*, *Acidobacteria*, and *Chloroflexi*, with the abundance of each phylum varying with the straw returning methods. For *Actinobacteriota*, the highest abundance was observed in the NT treatment (28.42%), with it being significantly higher than that in the NTS treatment (*p* < 0.05). For *Acidobacteria*, the NTS treatment results in the greatest abundance (20.23%), significantly higher than the NT and CT treatments. No significant differences were found in the abundances of *Proteobacteria*, *Gemmatimonadetes*, and *Chloroflexi* among all treatments, with the ranges of 20.68–24.70%, 16.21–21.39%, and 5.12–6.10%, respectively. These results suggested that NTS treatment increased the abundance of *Acidobacteria* in the soil, whereas NT treatment enhanced the abundance of *Actinobacteriota* (Figure 3a).

Based on the analysis of genus-level taxonomic diversity and the phylogenetic tree, soil bacteria subjected to different straw returning methods were predominantly composed of *Gemmatimonas*, Ellin6067, MND1, unidentified-SC-I-84, and unidentified- at the genus level. The abundance of MND1 in the NTS treatment was significantly higher than that in other cultivation patterns (*p* < 0.05), whereas the abundance of unidentified-SC-I-84 in the CT treatment was significantly higher than that in the NTS, NT, and DT treatments (*p* < 0.05). No significant differences were observed in the abundances of *Gemmatimonas*, Ellin6067, and unidentified-Gemmatimonadaceae among all treatments, with the ranges of 6.73–10.01%, 2.86–3.52%, and 1.71–2.30%, respectively (Figure 3b,c).

A Venn diagram was plotted based on the OTU classification results (Figure 3d). A total of 180 OTUs were shared by the NTS, NT, DT, and CT treatments. The NTS treatment produced 48 unique OTUs, accounting for 26.67% of the total OTUs; the NT treatment produced 38 unique OTUs, accounting for 21.11%; the DT treatment produced 15 unique OTUs, accounting for 8.33%; and the CT treatment produced 13 unique OTUs, accounting for 7.22%. The results indicated that the NTS treatment exhibited the largest number of unique OTUs, whereas the CT treatment comprised the fewest.

### 2.5. β-Diversity Analysis and Statistical Tests of Bacterial Community Composition

**Figure 4:**
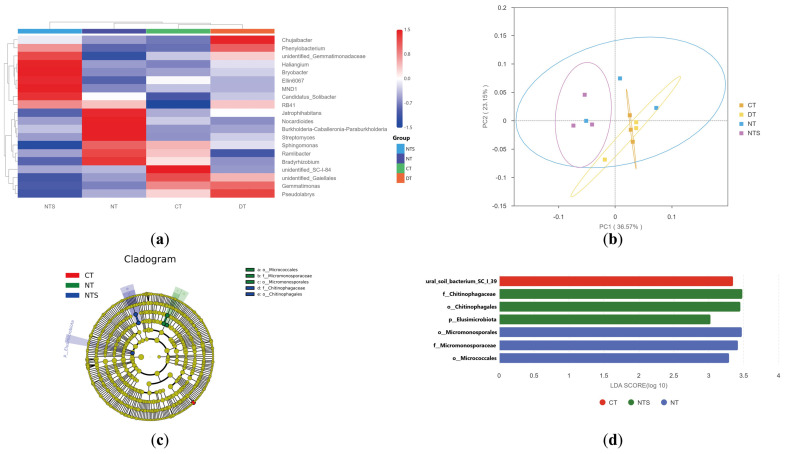
Clustering heatmap (**a**), PCoA plot (**b**), LEfSe phylogenetic tree (**c**), and LDA score distribution histogram (**d**) of soil bacteria in soybean fields under different straw returning patterns. The circles of different colors represent the confidence intervals for different treatments.

To identify the relative abundance of bacterial species across all treatments, the top 20 genera with the highest relative abundances were selected based on the species annotation and abundance data at the genus level for each sample, and a cluster analysis was performed at the species level to generate a heatmap (Figure 4a). The different straw returning methods exerted a significant effect on the dominant soil bacterial species in soybean fields. The dominant soil bacterial species in the NTS treatment included *Candidatus_Solibacter*, MND1, Ellin6067, *Bryobacter*, and *Haliangium*; those in the NT treatment included *Jatrophihabitans*, *Nocardioides*, *Streptomyces*, and *Ramlibacter*. In contrast, the CT and DT treatments contained fewer dominant bacterial species: only SC-I-84 and *Gaiellales* were dominant in the CT treatment, whereas only *Pseudolabrys*, *Phenylobacterium* and *Chujaibacter* were dominant in the DT treatment.

The results of principal coordinate analysis (PCoA) based on the Bray–Curtis distance algorithm for soil bacteria subjected to different straw returning methods are presented in Figure 4b. The first principal coordinate (PC1) explained 36.57% of the total variation in bacterial communities, and the second principal coordinate (PC2) explained 23.15%, with the first two principal coordinates collectively accounting for 59.72% of the total variation. PCoA results revealed that the soil bacterial community composition in the NTS treatment was significantly different from that in the CT and DT treatments. In contrast, the bacterial communities of the CT and DT treatments exhibited clustered overlap, indicating a high degree of similarity in soil bacterial community composition with no significant difference between the two treatments.

Significant differences were observed in the soil bacterial communities of the NTS, NT, and CT treatments in the phylogenetic tree (Figure 4c). Linear discriminant analysis effect size (LEfSe) with an LDA threshold of three was additionally performed to identify the significantly discriminant bacterial taxa, which included one phylum, three orders, two families, and one species (Figure 4d). Specifically, the soil bacterial community in the NTS treatment exhibited significantly higher abundances of *Elusimicrobiota* (phylum), *Chitinophagales* (order), and *Chitinophagaceae* (family) compared with other treatments; the NT treatment exhibited significantly higher abundances of *Micrococcales*, *Micromonosporales* (order), and *Micromonosporaceae* (family); the CT treatment exhibited a significantly higher abundance of Soil_bacterium_SC_I_39 (species); and no significantly discriminant taxa were identified in the DT treatment relative to the other treatments.

### 2.6. Functional Dynamics Analysis of Soil Bacterial Communities

**Figure 5:**
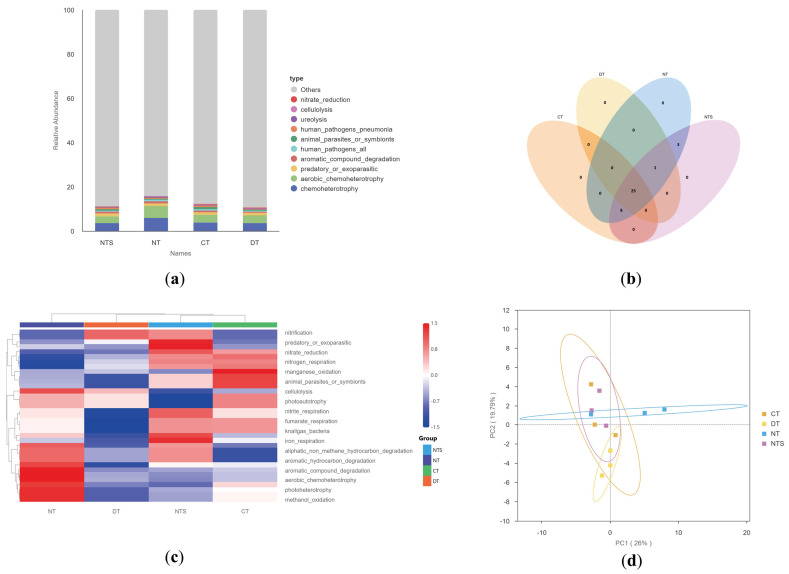
Bar plot of the relative abundance of bacterial community functions (**a**), Venn diagram (**b**), clustering heatmap (**c**), and PCoA analysis (**d**) in soybean fields under different straw returning patterns.

Functional prediction of soil bacterial communities was performed using FAPROTAX, and the functional groups with high relative abundance are shown in Figure 5. Among all treatments, chemoheterotrophy, aerobic chemoheterotrophy, predatory or exoparasitic, and aromatic compound degradation exhibited relatively high relative abundances (Figure 5a). The Venn diagram revealed that 28 functional groups were shared by all treatments, with no unique functional groups in any single treatment. Three functional groups were shared exclusively by the NTS and NT treatments, and thirty-three functional groups were shared by the NTS, NT, and CT treatments (Figure 5b). The dominant functional groups in the NT treatment included aromatic hydrocarbon degradation, aromatic compound degradation, photoheterotrophy, methanol oxidation, and aerobic chemoheterotrophy; those in the DT treatment included nitrification and predatory or exoparasitic; the dominant functional groups in the NTS treatment were predatory or exoparasitic, nitrite reduction, iron respiration, Knallgas bacteria, and nitrite respiration; and the dominant functional groups in the CT treatment were manganese oxidation and animal parasites or symbionts. In comparison, the NTS and NT treatments comprised more dominant functional groups, whereas the CT and DT treatments contained fewer (Figure 5c). Our principal coordinate analysis (PCoA) results indicated that the first principal coordinate (PC1) explained 26% of the functional variation, and the second principal coordinate (PC2) explained 19.79%, with the first two principal coordinates collectively accounting for 45.79% of the total variation (Figure 5d).

### 2.7. Correlation Analysis of Environmental Factors

**Figure 6:**
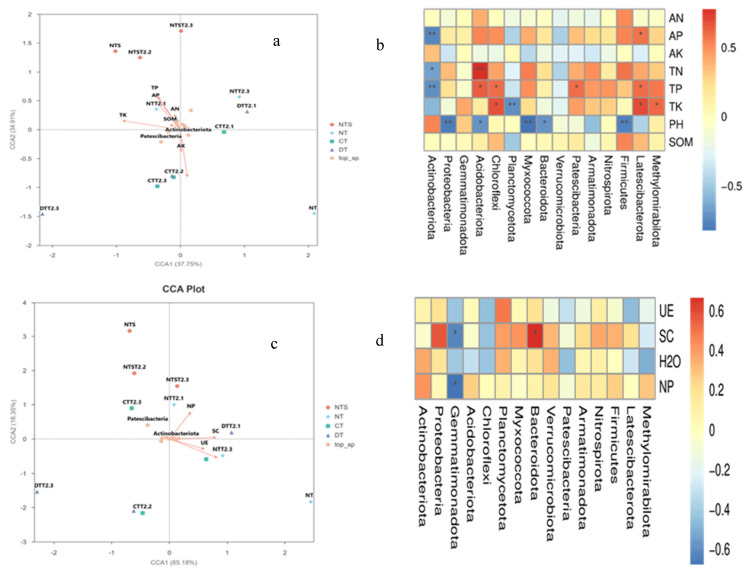
Canonical correspondence analysis (CCA) plots (**a**,**c**) and correlation heatmaps (**b**,**d**) between soil environmental factors and soil bacterial communities. * indicates a signiffcant level at *p* < 0.05, ** indicates a signiffcant level at *p* < 0.01.

Canonical correspondence analysis (CCA) was applied at the phylum level to further explore the key environmental driving factors of soil microorganisms (Figure 6a,c). The results showed that soil physicochemical properties were important factors with a significant impact on soil microorganisms. The first and second principal axes explained 37.75% and 34.91% of the total variation, respectively, with the first two principal axes collectively accounting for 72.66% of the total variation. Regarding the impact of soil enzymes on soil microorganisms, the first and second principal axes explained 65.18% and 16.35% of the total variation, respectively, and the cumulative explanatory rate of the first two principal axes reached 81.53%. Correlation analysis between soil properties and dominant bacterial phyla revealed that *Actinobacteriota* was extremely significantly negatively correlated with soil available phosphorus and total phosphorus and significantly negatively correlated with soil total nitrogen; *Proteobacteria*, *Myxococcota*, and *Firmicutes* were extremely significantly negatively correlated with soil pH; *Acidobacteriota* was extremely significantly positively correlated with soil total nitrogen and significantly positively correlated with soil total phosphorus; *Chloroflexi* was significantly positively correlated with soil total phosphorus and total potassium; *Planctomycetota* was extremely significantly negatively correlated with soil total potassium; *Acidobacteriota* and *Bacteroidota* were significantly negatively correlated with soil pH; *Patescibacteria* was significantly positively correlated with soil total phosphorus; *Latescibacterota* was significantly positively correlated with soil alkali-hydrolyzable nitrogen, total phosphorus, and total potassium; *Methylomirabilota* was significantly positively correlated with soil total potassium; *Gemmatimonadota* was significantly negatively correlated with soil invertase and acid phosphatase; and *Bacteroidota* was significantly positively correlated with soil invertase (Figure 6b,d).

## 3. Discussion

Based on the corn–soybean rotation system, the long-term field experiment was conducted to investigate the effects of NTS, NT, DT, and CT on soil fertility, soil enzyme activities, and soil bacterial community structure in the subsequent soybean field. The results showed that straw returning significantly increased the TP content in the plow layer of the subsequent soybean field compared with the CT treatment, potentially attributed to the input of organic phosphorus into the soil during straw decomposition [20,21]. Among the different straw returning methods, NTS exhibited the most pronounced effect, with the contents of TN, TP, and AP in the plow layer all higher than those in the other treatments. Notably, the AP content increased by 108.15% compared with the CT, which may be explained by two factors: first, low-molecular-weight organic acids are released during straw decomposition, which activate fixed phosphorus by chelating aluminum (Al^3+^), iron (Fe^3+^), and calcium (Ca^2+^) ions in the soil [22,23,24]; second, straw decomposition can stimulate the proliferation of phosphate-solubilizing microbial communities, thereby promoting the dissolution of inorganic phosphorus and the mineralization of organic phosphorus in the soil and further driving the transformation of stable phosphorus pools into available phosphorus pools in the soil [25,26,27]. Moreover, the NTS treatment reduced soil disturbance and nutrient leaching loss, further promoting nutrient accumulation, consistent with the findings of increased levels of soil nutrients in a long-term mulching experiment by Akhtar et al. and Zhong et al. [28,29].

Regarding the effects of straw returning on soil enzymes, our results indicated that soil enzyme activities in all straw returning treatments were higher than those in the CT treatment [30]. Similar findings were reported by Wu et al. [31] and Kashif et al. [32], who found that straw returning increased the activities of soil urease, phosphatase, and invertase, with their activities in the 0–10 cm soil layer increasing by 15.1%, 11.0%, and 88.4%, respectively, and soil enzyme activities showing a decreasing trend with the increase in soil depth. Among the different straw returning methods, the activities of soil urease, acid phosphatase, invertase, and catalase were the highest in the NTS treatment, followed by NT and DT, and the lowest in the CT treatment. These findings suggested that the NTS treatment exhibited stronger nitrogen and phosphorus transformation efficiency and organic matter degradation capacity in the soil plow layer [28,33]. Urease is involved in soil nitrogen mineralization, and acid phosphatase promotes the release of organic phosphorus; the improvement of their activities directly optimized the nutrient supply conditions for soybeans [34]. In addition, the high activities of invertase and catalase reflected the enhancement of soil organic matter decomposition and antioxidant stress capacity [35,36]. In contrast, the CT treatment exhibited generally low soil enzyme activities due to insufficient carbon sources caused by straw removal, which further confirmed the positive regulatory effect of straw returning on soil biological functions [37].

Bacterial diversity is an important indicator of soil ecosystem stability, and its community composition directly affects the efficiency of nutrient cycling. In this study, the Chao1, Shannon, and Observed_features indices were the highest in the NTS treatment, with the Chao1 and Shannon indices being 2.09 times and 1.10 times higher than those in the CT treatment, respectively, indicating that NTS could maximize the bacterial richness and diversity in the soil plow layer. This result was consistent with those of Liu et al. and Zhao [38,39], who demonstrated that conservation tillage combined with straw returning could promote microbial proliferation by providing sufficient carbon sources and a stable microenvironment. In terms of gene abundance and community composition, NTS significantly increased the abundance of *Acidobacteriota* (20.23%) at the phylum level, whereas NT enhanced the abundance of *Actinobacteriota* in the soil. Researchers have also found that tillage systems and straw returning methods have significant effects on bacterial community composition [40,41,42]. As important decomposers in the soil, *Acidobacteriota* and *Actinobacteriota* can decompose complex organic substances into simple compounds and release nutrients such as N, P, and K for plant absorption and utilization, potentially explaining why soil nutrient contents in straw returning treatments were higher than those in non-straw returning treatments [43,44].

Significant differences were observed in OTUs, dominant bacterial species, and phylogenetic branches among the NTS, NT, and CT treatments. The number of unique OTUs in the NTS treatment accounted for 26.67% of the total OTUs, a figure much higher than that in the other treatments; the number of dominant bacterial species in the NTS and NT treatments was significantly higher than that in the CT and DT treatments. Our PCoA and LEfSe analysis results also showed that the bacterial community in the NTS treatment was significantly different to that in the DT and CT treatments, with the community clustering of CT, DT, and NT overlapping. This finding may be attributed to the continuous carbon source input provided by straw mulching and the local microenvironment it provides, which promotes the formation of a unique bacterial community structure. In contrast, deep plowing and conventional tillage failed to form stable community differentiation due to uneven straw distribution or straw removal [45,46].

Analysis of bacterial community functions is beneficial in clarifying the relationship between straw returning and the bacterial system. FAPROTAX functional prediction showed that basic metabolic functions, such as chemoheterotrophy and aerobic chemoheterotrophy, accounted for the highest proportion in all treatments, which was consistent with the core functional characteristics of bacterial communities in most farmland soils [45]. Chemoheterotrophy and aerobic chemoheterotrophy are typical heterotrophic metabolic functions that rely on the decomposition and utilization of organic carbon; straw returning promoted the development of beneficial bacterial functional groups by inputting organic carbon [47]. The NTS treatment significantly enriched functional groups, such as predatory or exoparasitic, and nitrite reduction, which are directly involved in straw degradation and nitrogen cycling processes, further supporting the microbiological mechanism of soil fertility improvement using this approach [48]. The NT treatment was dominated by functions such as aromatic hydrocarbon degradation and methanol oxidation, indicating that straw returning may alter the utilization pattern of soil carbon sources, and was also related to the specific degradation of residual organic matter from straw stubble [49]; the CT treatment was enriched in functions related to manganese oxidation and animal parasitism, suggesting enhanced soil pollution remediation and structural improvement capacity, which promoted the synergy between soil fauna and microorganisms and was conducive to the stability of soil ecosystems, but also implied that conventional tillage might increase the risk of soil microecology [50].

Correlation analysis of environmental factors indicated that soil TN, TP, and AP were the key factors driving the changes in bacterial community structure. *Acidobacteriota* was extremely significantly positively correlated with TN and TP, whereas *Actinobacteriota* was significantly negatively correlated with AP and TN. This correlation pattern revealed the selective effect of nutrient contents on functional bacterial communities—high-nutrient environments were more conducive to the growth of oligotrophic bacteria such as *Acidobacteriota*, whereas *Actinobacteriota* exhibited a competitive advantage in relatively nutrient-deficient environments [51]. The results of correlation analysis between soil enzyme activities and bacterial communities showed that invertase activity was significantly positively correlated with the abundance of *Bacteroidota*, indicating that enzymes related to organic matter degradation and functional bacterial communities formed a synergistic network, which jointly promoted soil nutrient transformation.

## 4. Materials and Methods

### 4.1. Field Site

The above experiment was performed at the long-term monitoring experimental station for tillage methods in Chaoyangpo Town, Gongzhuling City, Jilin Province (124°45′46.74″ E, 43°36′19.71″ N). The soil is classified as thin typical black soil, with the parent material being Quaternary loess-like sediments. The experiment was established in 2017. The initial basic physical and chemical properties of the soil were as follows: pH 7.6; contents of organic matter, total nitrogen, total phosphorus, and total potassium: 22.8, 1.4, 0.6, and 18.4 g·kg^−1^, respectively; contents of available phosphorus and available potassium: 11.8 and 158.0 mg·kg^−1^, respectively; bulk density: 1.2 g·cm^−3^. The monitoring station is located on flat terrain with an altitude of 220 m, belonging to a temperate continental monsoon climate. The annual precipitation in the region is 450–650 mm, the annual average temperature is 4–5 °C, and the annual accumulated temperature is 2800 °C.

### 4.2. Experimental Design

Under the corn–soybean rotation system, a large-plot experimental design was adopted in this study, with four treatments established: no-tillage with straw mulching (NTS), no-tillage with stubble retention (NT), deep plowing with straw incorporation (DT), and conventional combined tillage (CT). Each treatment included 20 ridges, with a ridge length of 200 m, row spacing of 0.63 m, and a plot area of 2520 m^2^. NTS: After mechanical harvesting of the previous corn crop, the straw was crushed and evenly mulched on the soil surface, and sowing was conducted directly using a no-tillage seeder with straw mulching. NT: The aboveground part of the previous corn crop was harvested and the straw was removed from the field, with root stubble retained at roughly 10 cm; sowing was conducted directly using a Jilin Kangda 2BMQF-4 no-tillage air-blowing precision fertilizer seeder. DT: After the mechanical harvesting of the previous corn crop, the straw was crushed and plowed into the soil for ridging with a plowing depth of 25–30 cm, and the sowing method was identical to that of the NT approach. CT: Corn was harvested mechanically, the straw was removed from the field, conventional ridging was performed, and the sowing method was identical to that of the NT treatment. The maize straw used for incorporation comprised an average moisture content of 23%, particle size of 8–10 cm, ash content of 7.8%, total carbon content of 432 g·kg^−1^, total nitrogen content of 9.3 g·kg^−1^, total phosphorus content of 3.6 g·kg^−1^, C/N ratio of 42, cellulose content of 38%, hemicellulose content of 26%, and lignin content of 17%.

The cropping system comprised annual maize–soybean rotation starting in 2017. Maize was sown annually in late April and harvested in early October. The maize cultivar Nonghua 101 was employed, with a planting density of 60,000 plants·hm^−2^ in single rows on ridges. The soybean cultivar Jiyu 86 was utilized, with a planting density of 250,000 plants·hm^−2^ in double rows on ridges. Soybean was sown in early May and harvested in late September. Maize-specific fertilizer was applied at 750 kg·hm^−2^ (N:P:K = 25:18:12) and soybean-specific fertilizer at 300 kg·hm^−2^ (N:P:K = 15:15:15). All fertilizers were band-applied into the soil during sowing. All treatments were strictly implemented based on the experimental design, with unified field management and pest control.

### 4.3. Sample Collection

In 2024, soil samples from the 0–20 cm soil layer were collected from the four treatments at the soybean filling stage. The “S” sampling method was used, with 5 sampling points selected in each treatment plot and mixed into one composite sample; three replicates were set for each treatment. Soil samples were passed through a 2 mm sieve to remove impurities such as leaves, roots, and stones and then divided into two parts: one part was air-dried naturally at room temperature and ground for the determination of soil physical and chemical properties; the other part comprised fresh soil stored in a refrigerator at −80 °C for the determination of soil enzyme activities and microbial sequencing analysis. The other portion of fresh soil was immediately frozen in liquid nitrogen and stored at −80 °C for the determination of soil enzyme activities and microbial sequencing. The storage duration did not exceed 72 h, avoiding repeated freeze–thaw cycles. All physicochemical properties and DNA extractions were analyzed in duplicate for each soil sample to ensure analytical accuracy.

### 4.4. Soil Properties and Enzyme Activities

Soil physical and chemical properties were determined based on the Technical Specification for Soil Analysis [52]. Soil total nitrogen content was determined using an elemental analyzer (Elementarvario MACRO cube, Germany). Alkali-hydrolyzable nitrogen content was measured using the alkali hydrolysis diffusion method. Both available phosphorus and total phosphorus contents were determined using the molybdenum–antimony anti-colorimetric method. Available potassium content was measured using the ammonium acetate extraction–flame photometer method. Total potassium content was determined using the NaOH alkali fusion–flame photometry method. Soil pH value was measured using the potentiometric method (Model of the pH meter HD-PT1, water:soil = 2.5:1). Soil organic matter (SOM) content was determined using the potassium dichromate volumetric method with external heating. Soil enzyme activities were determined according to the method of Chen et al. [53]. Soil invertase (INV) activity was measured using the 3,5-dinitrosalicylic acid colorimetric method. Soil catalase (CAT) activity was determined using the potassium permanganate titration method. Lastly, soil urease (URE) activity was measured using the sodium phenoxide–sodium hypochlorite colorimetric method.

### 4.5. Soil DNA Extraction and PCR Amplification

Soil DNA from the different samples was extracted based on the instructions of the E.Z.N.A.^®^ Soil DNA Kit (D5625-02, Omega, Norcross, GA, USA). The quality of extracted DNA was detected by means of 1% agarose gel electrophoresis, and the DNA concentration and purity were determined using a NanoDrop 2000 spectrophotometer (Thermo Fisher Scientific, Wilmington, DE, USA). The DNA samples were stored at −20 °C until further analysis. The V3–V4 hypervariable regions of the bacterial 16S rRNA gene were amplified using the primers 515F (5′-GTGCCAGCMGCCGCGGTAA-3′) and 806R (5′-GGACTACHVGGGTWTCTAAT-3′) [54]. Each PCR mixture contained 15 µL of Phusion High-Fidelity PCR Master Mix, 0.2 µM of each primer, and 10 ng of genomic DNA template. The PCR procedure was as follows: initial denaturation at 98 °C for 1 min, followed by 30 cycles of denaturation at 98 °C for 10 s, annealing at 50 °C for 30 s and extension at 72 °C for 30 s, with a final extension at 72 °C for 5 min. After purification and quantitative analysis of the PCR amplicons, high-throughput sequencing was performed by Beijing Novogene Bioinformatics Technology Co., Ltd., Beijing, China.

### 4.6. Bioinformatic Analysis

Raw sequencing data were quality-controlled using Fastp 0.23.1 [55] and spliced using FLASH 1.2.11 [56]. Clustering and chimera removal were performed using the UPARSE algorithm in USEARCH11, and OTUs were obtained based on 97% sequence similarity. Taxonomic annotation of OTUs was conducted using RDP classifier 2.13 with the bacterial 16S rRNA gene database Silva138/16S_bacteria (https://www.arb-silva.de accessed on 12 January 2025) as the reference. Mothur1.30.2 was used to calculate the α-diversity indices. Principal coordinate analysis (PCoA) was performed based on the Bray–Curtis distance algorithm. Analysis of similarities (ANOSIM) was used to test the similarity of microbial community structures among samples, and permutational multivariate analysis of variance (PERMANOVA) was combined to analyze the significance of differences in microbial community structures among sample groups. Redundancy analysis (RDA) was used to explore the correlations between soil microorganisms and environmental factors. FAPROTAX [57] was applied to predict the functions of soil bacterial communities.

Excel 2016 was used for data collation, SPSS Statistics 24.0 was employed for one-way analysis of variance (ANOVA) with the least significant difference (LSD) test (*p* < 0.05) to analyze the differences among different treatments, and Origin2021 was used for drawing figures.

## 5. Conclusions

NTS was the optimal approach that simultaneously improved soil fertility and optimized bacterial communities. By combining straw mulching with no-tillage, this approach not only enhanced the nutrient supply effect of straw but also minimized soil disturbance to microbial habitats, forming a positive cycle of “organic matter accumulation–microbial proliferation–efficient nutrient transformation”. Although NT held an advantage in the abundance of *Actinobacteriota*, the improvement effects on soil nutrients and bacterial diversity were limited due to the lack of sufficient straw carbon sources; DT disrupted the stability of microbial communities due to severe soil disturbance, limiting the ecological benefits of straw returning; and CT led to low soil fertility and biological activity due to straw removal, making it difficult to meet the demand for high soybean yield. It is important to acknowledge the limitations of our study. For example, we focused solely on the analysis of soil bacterial communities at the soybean grain-filling stage and did not include fungi, which also play important roles in straw decomposition. In the future, we will focus on exploring the successional dynamics and synergistic interactions of bacterial and fungal communities across the entire soybean growth period. Moreover, long-term monitoring and effect evaluation of the NTS system on soybean yield stability under different climatic conditions will be conducted to clarify its adaptability and application potential under diverse climatic scenarios, including high temperature, low temperature, drought, and heavy rainfall. Such measures will provide more comprehensive and systematic theoretical support and a practical basis for the regional optimization and large-scale promotion of straw returning patterns under maize–soybean rotation systems in Northeast China.

## Figures and Tables

**Table 1:** Soil nutrients and soil pH under different tillage practices.

	NTS	NT	CT	DT
AN (mg/kg)	1.08 ± 0.12 a	0.98 ± 0.04 a	1.02 ± 0.04 a	1.02 ± 0.04 a
AP (mg/kg)	0.55 ± 0.12 a	0.41 ± 0.03 b	0.42 ± 0.02 b	0.42 ± 0.02 b
AK (mg/kg)	21.38 ± 1.13 a	21.44 ± 0.74 a	21.69 ± 0.81 a	21.86 ± 0.24 a
TN (g/kg)	142.49 ± 19.45 a	117.99 ± 10.00 b	120.96 ± 3.64 b	119.24 ± 6.55 b
TP (g/kg)	41.30 ± 14.34 a	22.82 ± 7.28 b	19.84 ± 1.12 c	22.46 ± 3.39 b
TK (g/kg)	165.33 ± 12.06 a	158.67 ± 15.63 a	159.00 ± 8.19 a	161.67 ± 14.22 a
PH	5.62 ± 0.08 ab	5.60 ± 0.14 ab	5.41 ± 0.18 b	5.69 ± 0.08 a
SOM (g/kg)	17.40 ± 1.87 a	15.93 ± 0.78 a	17.67 ± 1.22 a	16.30 ± 0.95 a

Different lowercase letters in the same line indicate a significant difference between different groups (*p* < 0.05). SOM: Soil organic matter; AP: Available phosphorus; AK: Available potassium; AN: Alkali-hydrolyzable nitrogen; TN: total nitrogen; TP: total phosphorus; TK: total potassium.

## Data Availability

The original contributions presented in this study are included in the article. Further inquiries can be directed to the corresponding authors.
